# Factors associated with anemia among adolescent girls in Western India: insights from a multi-centric cross-sectional study

**DOI:** 10.3389/fgwh.2026.1793809

**Published:** 2026-05-18

**Authors:** Krupal Joshi, Harsh Bakshi, A. M. Kadri, Ashwini Agarwal, Astha Vala, Sagar Dholariya, Amit Sonagra, Manisha Upadhyay, Garima Anandani, Gyanendra Singh, Parth Goswami

**Affiliations:** 1 All India Institute of Medical Sciences, Rajkot, India; 2 GMERS Medical College-Sola, Ahmedabad, India; 3 State Health System Resource Centre-Gujarat, Gandhinagar, India

**Keywords:** adolescent, anemia, determinants of anemia, India, multicentric cross-sectional study

## Abstract

**Background:**

Anemia remains a persistent public health challenge among adolescent girls in India, with states like Gujarat continuing to report a high burden despite ongoing national nutrition and anemia control programs. Regional disparities, particularly across rural and tribal populations, further exacerbate the problem. This study aimed to estimate the prevalence of anemia among adolescent girls in Gujarat, India, and examine its sociodemographic, nutritional, behavioral, hematological, and biochemical associated factors.

**Methods:**

A community-based, multi-centric cross-sectional study was conducted among 2,815 adolescent girls in ten purposively selected districts of Gujarat, representing rural, urban, and tribal settings. A multistage Probability Proportionate to Size sampling strategy was used. Data collection included structured interviews, anthropometric assessments, and venous blood sampling. Hematological parameters were analyzed using an automated six-part hematology analyzer, while biochemical markers—including iron profile, ferritin, vitamin B12, folate, albumin, prealbumin, and C-reactive protein (CRP)—were measured using standardized laboratory platforms. Anemia was defined according to World Health Organization criteria. Statistical analyses were performed using descriptive statistics and inferential tests with a significance level of *p* < 0.05.

**Results:**

The overall prevalence of anemia was 60.85%, with marked inter-district variation ranging from 42.37% to 75.00%. Mild anemia was most prevalent (33.46%), followed by moderate (25.04%) and severe anemia (2.34%). Anemia prevalence was significantly higher among tribal (73.57%) and rural (56.39%) adolescents compared to urban counterparts (48.19%) (*p* < 0.001). Socioeconomic disadvantage, lack of digital access, poor knowledge regarding anemia, and non-consumption of iron–folic acid (IFA) supplements were significantly associated with anemia. A strong inverse association was observed between anemia prevalence and knowledge scores (*p* < 0.001). Multivariate regression analysis demonstrated significant association between health insurance coverage and anemia prevalence (aOR = 1.15, *p* = 0.004). Biochemical analysis revealed significantly lower serum iron, ferritin, transferrin saturation, albumin, and prealbumin levels among anemic girls, alongside elevated total iron-binding capacity and CRP, indicating a predominance of iron deficiency anemia with an inflammatory component. Hemoglobinopathy screening identified beta-thalassemia trait (5.44%) and sickle cell trait (4.80%), highlighting regional genetic contributors.

**Conclusions:**

Anemia was highly prevalent among adolescent girls in Gujarat, affecting 60.85% of participants, with higher burden observed in tribal and rural populations. Significant associations were identified with socioeconomic status, health insurance coverage, knowledge levels, and iron–folic acid supplementation. Biochemical findings indicated iron deficiency with an inflammatory component, and hemoglobinopathy traits were also present among a subset of participants. These findings highlight the multifactorial nature of anemia in the study population.

## 1 Introduction

Anemia and malnutrition continue to pose substantial public health challenges across the globe, particularly affecting women of reproductive age and adolescents. According to the Global Burden of Disease (GBD) Study 2023, anemia affects an estimated 1.8 billion individuals worldwide, making it one of the most prevalent nutritional deficiencies globally (1). Adolescent girls remain disproportionately impacted, with global anemia prevalence estimated at 29.9%, driven primarily by iron deficiency, menstrual blood loss, dietary inadequacies, infections, and gender-related inequities in access to nutrition (2). Malnutrition, encompassing both undernutrition and overweight/obesity, also remains a persistent challenge. In 2023, 148 million children globally were stunted, and 45 million were wasted, reflecting ongoing disparities in nutritional access and health systems performance (3).

In India, the burden of anemia is among the highest globally. The National Family Health Survey-5 (NFHS-5, 2021) reported anemia prevalence of 57% among women of reproductive age and 59% among adolescent girls aged 15–19 years (4). India contributes to nearly 40% of the global anemia burden among adolescent girls, underscoring the magnitude and persistence of the problem (5). Dietary iron inadequacy, poor iron absorption due to phytate-rich diets, early marriage, poor menstrual hygiene, intestinal parasitic infections, and limited adherence to iron–folic acid (IFA) supplementation programs are critical determinants (6). Simultaneously, India faces the dual burden of malnutrition, with 35.5% stunting, 19.3% wasting, and a steadily rising prevalence of overweight among adolescents and women, particularly in urban areas (4). These complex and overlapping nutrition challenges call for district-specific, evidence-driven approaches to inform policy and programming.

Gujarat, despite being one of India's fastest-growing states in economic and infrastructural development, continues to face a significant nutrition deficit. As per NFHS-5, 59% of adolescent girls and 67% of pregnant women in Gujarat are anemic, rates that remain higher than many other Indian states (4). Undernutrition persists, with 35% stunting and 15% wasting reported among children under five (4). Moreover, findings from the Comprehensive Nutrition Survey of Gujarat (CNSG, 2019) highlight pronounced rural and tribal–urban disparities, particularly high anemia prevalence in tribal districts, attributed to dietary monotony, limited access to health services, and food insecurity (7). Urban regions, meanwhile, are experiencing rising tendencies toward overweight and obesity, reflecting a shifting nutritional profile and emerging risk of noncommunicable diseases (8). These patterns indicate that economic development alone is insufficient to address entrenched nutritional vulnerabilities without targeted, population-specific interventions.

Adolescence represents a critical developmental window during which nutritional deficiencies—especially anemia—can have immediate and long-term consequences. Iron deficiency during adolescence impairs cognitive performance, immunity, physical productivity, and, in females, increases the risk of adverse outcomes in future pregnancies (9). National initiatives such as Anemia Mukt Bharat (AMB) and POSHAN Abhiyaan aim to address anemia through IFA supplementation, dietary diversification, deworming, and behavior-change communication. However, the lack of district-level biochemical and dietary data limits the creation of precise, context-specific interventions for adolescent girls (10).

Despite the availability of national datasets such as NFHS-5 and periodic state surveys, there remains a critical gap in granular, district-level, biochemically validated, and diet-specific data on anemia among adolescent girls in Gujarat. Existing surveys predominantly rely on hemoglobin estimation and broad sociodemographic indicators, offering limited insight into the biochemical determinants, dietary patterns, iron bioavailability, and region-specific risk factors that drive the persistently high anemia burden. Moreover, adolescent girls represent a nutritionally vulnerable group whose health outcomes directly influence future maternal health, birth outcomes, and intergenerational cycles of malnutrition. The heterogeneity observed across Gujarat—particularly the substantial disparities between tribal, rural, and urban districts—further underscores the need for localized evidence to guide targeted interventions rather than one-size-fits-all strategies. While national programs such as Anemia Mukt Bharat advocate multi-component anemia control strategies, their effectiveness is constrained by the lack of district-level operational data to tailor implementation. Therefore, a comprehensive assessment integrating biochemical parameters, dietary intake, and contextual sociodemographic factors is essential to inform precision public health planning, strengthen existing state nutrition programs, and contribute to India's progress toward global anemia reduction targets. This study aims to fill these knowledge gaps by generating robust, district-specific evidence that can directly support policymakers, program managers, and public health practitioners in designing effective, culturally appropriate, and context-sensitive interventions for adolescent girls across Gujarat.

## 2 Materials and methods

### 2.1 Study design and setting

This community-based cross-sectional study was conducted to assess the prevalence and determinants of anemia and malnutrition among adolescent girls aged 10–19 years in Gujarat, India. Ten districts—Anand, Dahod, Jamnagar, Mehsana, Navsari, Rajkot, Sabarkantha, Surat, Tapi, and Vadodara—were purposively selected to capture the diversity of rural, urban, and tribal populations within the state. These districts represent varied socio-economic and nutritional environments, allowing for comprehensive state-level inference. The study was implemented through district-level field teams who adhered to standardized protocols to ensure procedural uniformity across all regions.

### 2.2 Sampling strategy

A multistage sampling approach using Probability Proportionate to Size (PPS) was adopted to obtain a representative sample. In the first stage, a complete list of Primary Health Centres (PHCs) within the ten selected districts was obtained from the Gujarat Health Department. Districts were stratified based on their predominant demographic characteristics—rural, urban, or tribal—and PHCs were chosen proportionate to the population served in each stratum. In the second stage, households in the selected PHC catchment areas were enumerated, and eligible adolescent girls were selected through simple random sampling. Household listings within the selected PHC catchment areas were obtained using population registers maintained at the respective Primary Health Centres and through household enumeration conducted by field investigators. During enumeration, all households were visited and eligible adolescent girls aged 10–19 years were identified and listed to create a sampling frame. From this sampling frame, participants were selected using simple random sampling. This multilevel selection process ensured adequate representation of adolescents across the range of geographical and socio-cultural contexts.

### 2.3 Sample size estimation

The required sample size was calculated based on an anticipated anemia prevalence of 50% among adolescent girls as per the NFHS-5 report (4), with 10% relative precision, a 95% confidence interval, and a design effect of 2 to account for cluster sampling. This calculation yielded a target of 280 adolescent girls per district, resulting in a planned total sample size of 2,815 participants across all ten districts.

### 2.4 Eligibility criteria

Eligible participants included adolescent girls aged 10–19 years who had been residing in the selected district for at least six months and were able to provide informed assent/consent, with parental consent obtained for those under 18 years. Participants were required to agree to anthropometric assessment, dietary evaluation, and venous blood sampling. Exclusion criteria included adolescents who had migrated to the district within the last six months, refusal to participate or provide blood samples, and those with known chronic medical conditions that could affect hematological parameters, such as chronic kidney disease, active tuberculosis, or malignancies.

### 2.5 Operational definitions

Anemia was defined using World Health Organization (WHO) hemoglobin cut-offs for adolescent females, with mild anemia classified as hemoglobin 11.0–11.9 g/dL, moderate anemia as 8.0–10.9 g/dL, and severe anemia as <8.0 g/dL (2). Nutritional status was assessed using BMI-for-age Z-scores based on WHO growth reference standards. These operational definitions ensured consistency and comparability with global studies on adolescent nutritional health.

### 2.6 Data collection procedures

#### 2.6.1 Interview and socio-demographic assessment

A pretested structured questionnaire was administered by trained female field investigators to collect information on sociodemographic characteristics, menstrual history, dietary habits, and health service utilization. The questionnaire was initially developed in English and translated into Gujarati, followed by back-translation to ensure linguistic accuracy. The tool was pilot tested among adolescent girls in a non-study district to assess clarity and comprehension. Internal consistency of the anemia knowledge scale was assessed using Cronbach's alpha, which demonstrated acceptable reliability (α = 0.78).

#### 2.6.2 Anthropometric measurements

Anthropometric assessments included measurements of height and weight using portable stadiometers and calibrated digital weighing scales. Measurements were taken following WHO standard protocols, with participants wearing light clothing and no footwear. BMI was calculated as weight (kg) divided by height squared (m²). Standardization sessions were conducted periodically, and supervisory teams performed random spot checks to ensure inter-observer reliability.

#### 2.6.3 Biochemical assessments

Biochemical data were obtained through venous blood samples (6–8 mL), collected under aseptic conditions into both plain and Ethylenediaminetetraacetic acid (EDTA) vacutainers (11). All laboratory analyses were conducted using standardized instruments and protocols (12). Hematological parameters were measured using the Sysmex XN-1000 six-part hematology analyzer (13), while biochemical assays were performed using the VITROS 5600 platform (14), employing dry chemistry and enhanced electrochemiluminescence methods. In addition to hemoglobin, a broad panel of indicators was evaluated, including hematological parameters, serum iron, total iron-binding capacity (TIBC), transferrin, and serum ferritin. Vitamin status was assessed by serum vitamin B12 and folate levels. Other nutritional markers included serum albumin, prealbumin, magnesium, and vitamin D. C-reactive protein (CRP) was measured as a indicator of inflammation, and hemoglobin variants were identified where indicated. Peripheral blood smears were prepared using Leishman and field staining techniques for morphological examination (15).

### 2.7 Quality assurance and field monitoring

Robust quality assurance mechanisms were integrated throughout the study. Ten district-level field teams consisting of trained data collector. Supervisory officers conducted regular monitoring visits to verify adherence to study protocols, ensure standardization of anthropometric techniques, and oversee the cold chain and safe transport of biological specimens. Data entry quality checks and laboratory validation processes were implemented routinely to maintain accuracy and reliability.

### 2.8 Ethical considerations

The study protocol was reviewed and approved by the Institutional Ethics Committee of AIIMS Rajkot (Approval No: AIIMS/RAJKOT/5th IEC/FB/37). Prior to participation, written informed consent was obtained from all participants aged 18 and above, and assent along with parental consent was obtained for those under 18 years. Confidentiality was maintained by assigning unique participant identifiers and securely storing all data. Blood sampling adhered to standard biosafety and infection control guidelines. Participation was voluntary, and individuals were free to withdraw at any point without any negative consequences.

### 2.9 Statistical analysis

Data were entered into Microsoft Excel 2013 and analyzed using Jamovi (version 2.5.3.0). Descriptive statistics, including means, standard deviations, frequencies, and proportions, were calculated to summarize participant characteristics and laboratory findings. The Chi-square test was used to assess differences in categorical variables, while independent sample *t*-tests and one-way ANOVA were employed for continuous variables, depending on distributional assumptions. Pearson correlation analysis was performed to examine associations between hematological parameters and nutritional biomarkers. Multivariate binary logistic regression analysis was performed to determine the independent predictors of anemia with inclusion variables of *p* < 0.05 in bivariate analysis. The final model was evaluated for adequacy using the Hosmer—Lemeshow goodness-of-fit test. Odds Ratio (OR), 95% Confidence Intervals (CI) were reported along with *p*-value. All statistical tests were two-tailed, with significance defined at *p* < 0.05.

## 3 Results

### 3.1 Demographic profile and anemia prevalence among study participants

**Figure 1:**
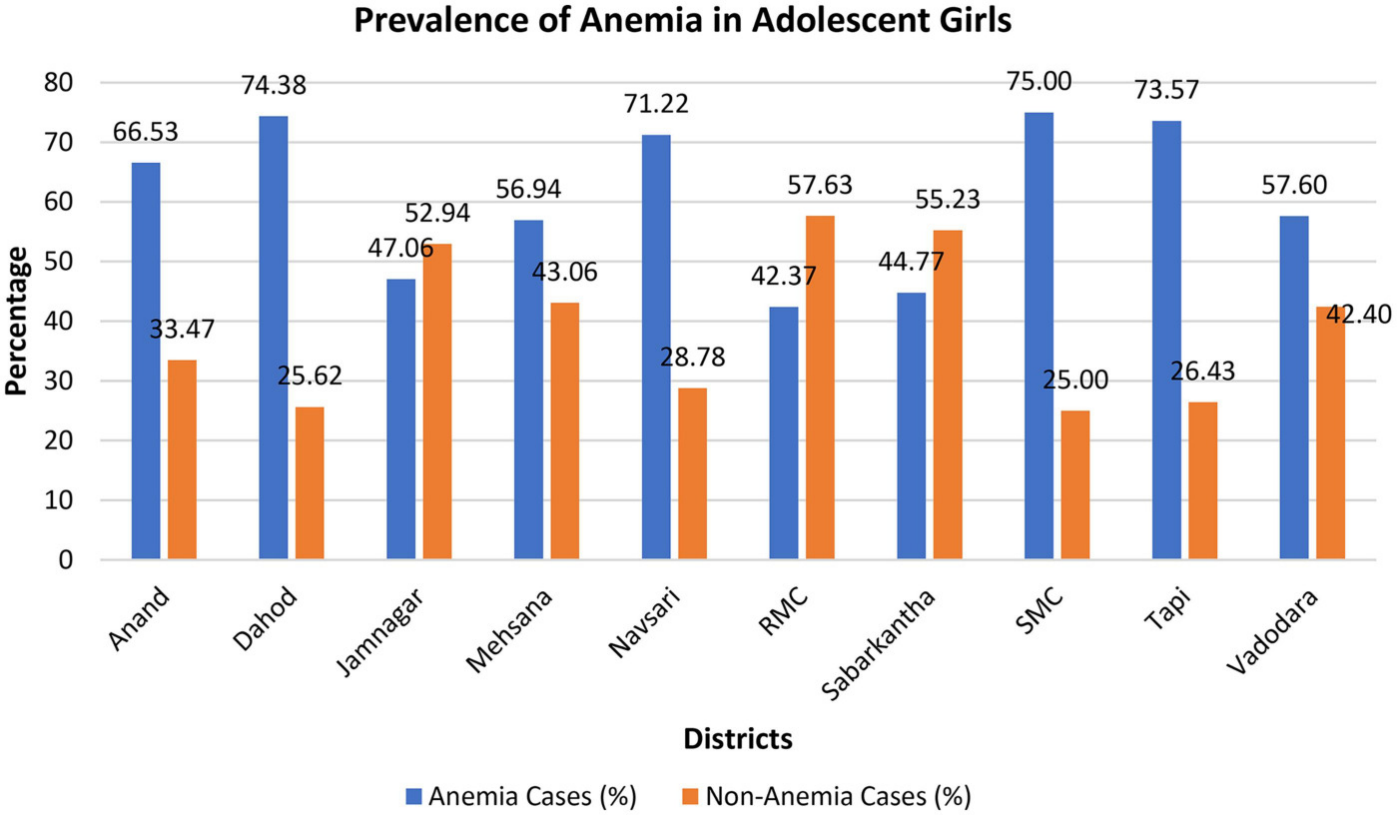
Prevalence of anemia in adolescent girls across different districts of Gujarat.

A total of 2,815 adolescent girls participated in the study. The majority were from rural areas (40.46%), followed by urban (30.37%) and tribal regions (29.17%). Most participants were aged 15–19 years (60.57%), and a large proportion had completed primary (41.78%) or secondary (36.51%) education. Nearly four-fifths (81.95%) were currently attending school, predominantly in government institutions (69.16%). Socioeconomic profiling revealed that 62.98% resided in pukka houses, 52.29% belonged to nuclear families, and 91.69% were Hindus. The participants primarily belonged to Other Backward Classes (33.61%) and Scheduled Tribes (27.89%). More than half (56.09%) held Below Poverty Line (BPL) cards, and 45.44% had some form of health insurance. Only a small proportion reported engagement in paid work (6.12%) or vocational training (11.86%). The overall prevalence of anemia among adolescent girls was 60.85%, indicating that more than half of the participants were anemic. The prevalence varied substantially across districts, ranging from 42.37% in Rajkot Municipal Corporation to 75.00% in Sabarkantha district. High-burden districts included Dahod (74.38%), Tapi (73.57%), and Navsari (71.22%), while lower prevalence was seen in Jamnagar (47.06%) and Surat Municipal Corporation (44.77%) (Figure 1). With respect to anemia severity, mild anemia was most common (33.46%), followed by moderate (25.04%) and severe anemia (2.34%). Only 39.15% of participants had normal hemoglobin levels, underscoring a considerable public health burden (Table 1).

**Table 1:** Distribution of anemia according to severity in adolescent girls.

Anemia Severity	Adolescent Girls (*N* = 2,815) *n* [%]
Normal anemia (Hb >12 g/dL)	1,102 (39.15%)
Mild anemia (Hb 11– < 11.9 g/dL)	942 (33.46%)
Moderate anemia (Hb 8– < 10.9 g/dL)	705 (25.04%)
Severe anemia (Hb < 8 g/dL)	66 (2.34%)

### 3.2 Prevalence of anemia by sociodemographic factors and nutritional status

A significant association was observed between area of residence and anemia prevalence (*p* < 0.001). The highest prevalence occurred among girls from tribal areas (73.57%), followed by rural (56.39%) and urban areas (48.19%). Anemia prevalence was significantly higher among girls belonging to BPL families (61.68%) compared to non-BPL families (56.57%) (*p* = 0.009). Similarly, those unsure about their health insurance coverage (65.86%) exhibited a higher prevalence than those who reported being insured (58.80%; *p* = 0.005). Lack of access to digital devices such as mobile phones or computers was also significantly associated with higher anemia prevalence (*p* = 0.01), suggesting that socioeconomic and resource-related inequalities influence anemia burden. No significant associations were found for age, education, type of family, religion, or caste (Table 2). Analysis of anthropometric parameters revealed no significant association between BMI category and anemia (*p* = 0.59). Although prevalence was marginally higher among underweight girls (61.52%), differences across weight groups were not statistically significant. Based on height-for-age z-scores (HAZ), 2.56% were stunted, and based on BMI-for-age z-scores (BAZ), 0.07% were underweight, while 12.1% were over-nourished, indicating a dual burden of malnutrition. Younger adolescents (10–14 years) had a higher proportion of stunting (3.4%) than older adolescents (15–19 years, 1.9%) (Supplementary Tables S1–S3—Supplementary Material).

**Table 2:** Association of location and socio-cultural classification with prevalence of Anemia in adolescent girls.

Variables	Anemia (*n*, row %, Col %)	Not anemia (*n*, Row %, Col %)	Raw total (*n*, %)	*P* value
Location and socio-cultural classification				
Rural	463 (56.4%, 27.0%)	358 (43.6%, 32.5%)	821 (29.2%)	**<0**.**001**
Tribal	838 (73.6%,48.9%)	301 (26.4%, 27.3%)	1,139 (40.5%)	
Urban	412 (48.19%, 24.1%)	443 (51.81%, 40.2%)	855 (30.4%)	
Column Total (*n*, %)	1,713 (60.9%)	1,102 (39.1%)	2,815 (100%)	
Age in years				
10–14 years	684 (61.6%, 39.9%)	426 (38.4%,38.7%)	1,110 (39.4%)	0.5
15–19 years	1,029 (60.3%,60.1%)	676 (39.6%,61.3%)	1,705 (60.6%)	
Column Total (*n*, %)	1,713 (60.8%)	1,102 (39.1%)	2,815 (100%)	
Educational level				
None	23 (50.0%,1.4%)	23 (50.0%, 2.2%)	46 (1.7%)	0.2
Primary education (up to 8th)	692 (61.0%, 41.6%)	443 (39.0%, 42.1%)	1,135 (41.8%)	
Secondary education (9th–10th)	623 (62.9%, 37.5%)	368 (37.1%, 35.0%)	991 (36.5%)	
Higher secondary education (11th–12th)	325 (59.9%, 19.5%)	218 (40.1%, 20.7%)	543 (20.0%)	
Column Total (*n*, %)	1,663 (61.2%)	1,052 (38.8%)	2,715 (100%)^a^	
Type of houses				
Pukka	1,066 (60.1%,62.2%)	707 (39.9%, 64.2%)	1,773 (62.9%)	0.5
Kutcha	338 (61.7%, 19.7%)	210 (38.3%,19.1%)	548 (19.5%)	
Semi-pukka	309 (62.5%,18.0%)	185 (37.4%,16.8%)	494 (17.5%)	
Column Total (*n*, %)	1,713 (60.8%)	1,102 (39.2%)	2,815 (100%)	
Type of Family				
Nuclear	1,066 (60.1%, 62.2%)	707 (39.9%, 64.2%)	1,773 (62.9%)	0.5
Extended	338 (61.7%, 19.7%)	210 (38.3%, 19.1%)	548 (19.5%)	
Joint	309 (62.5%, 18.0%)	185 (37.4%, 16.8%)	494 (17.5%)	
Column Total (*n*, %)	1,713 (60.85%)	1,102 (39.15%)	2,815 (100%)	
Religion				
Christianity	46 (54.1%,2.7%)	39 (45.9%, 3.4%)	85 (3.0%)	0.3
Hinduism	1,580 (61.2%, 92.2%)	1,001 (38.8%,90.8%)	2,581 (91.7%)	
Islam	87 (58.4%, 5.1%)	62 (41.6%, 5.6%)	149 (5.3%)	
Column Total (*n*, %)	1,713 (60.8%)	1,102 (39.1%)	2,815 (100%)	
Caste				
Don't know	270 (57.9%, 15.8%)	196 (42.1%, 17.8%)	466 (16.55%)	0.2
General	239 (62.4%, 13.9%)	144 (37.6%, 13.1%)	383 (13.6%)	
OBC	577 (60.9%, 33.7%)	369 (39.0%, 33.5%)	946 (33.6%)	
SC	133 (56.60%, 7.8%)	102 (43.4%, 9.3%)	235 (8.3%)	
ST	494 (62.9%, 28.8%)	291 (37.1%, 26.4%)	785 (27.8%)	
Column Total (*n*, %)	1,713 (60.8%)	1,102 (39.1%)	2,815 (100%)	
Family below poverty Line				
Don't know	313 (64.8%,18.3%)	170 (35.2%, 15.4%)	483 (17.2%)	**0**.**009**
No	426 (56.6%, 24.9%)	327 (43.4%,29.7%)	753 (26.7%)	
Yes	974 (61.7%, 56.7%)	605 (38.3%, 54.9%)	1,579 (56.09%)	
Column Total (*n*, %)	1,713 (60.8%)	1,102 (39.1%)	2,815 (100%)	
Health Insurance coverage				
Don't know	492 (65.9%, 28.7%)	255 (34.1%, 23.1%)	747 (26.5%)	**0**.**005**
No	469 (59.4%, 27.4%)	320 (40.6%, 29.0%)	789 (28.03%)	
Yes	752 (58.8%, 43.9%)	527 (41.2%, 47.8%)	1,279 (45.4%)	
Column Total (*n*, %)	1,713 (60.8%)	1,102 (39.1%)	2,815 (100%)	
Using mobile/computer/laptop/tablet				
Common device for entire family	490 (61.9%, 28.6%)	301 (38.1%, 27.3%)	791 (28.1%)	**0**.**01**
No	346 (65.4%, 20.2%)	183 (34.6%, 16.6%)	529 (18.8%)	
Yes	877 (58.66%, 51.20%)	618 (41.3%, 56.1%)	1,495 (53.1) %	
Column Total (*n*, %)	1,713 (60.8%)	1,102 (39.1%)	2,815 (100%)	

a Missing values in certain variables (e.g., some participants did not respond or selected “don't know”).

Bold value represents significant *p*-value.

### 3.3 Medical and nutritional factors associated with anemia

No statistically significant associations were observed between past medical or nutritional interventions and anemia prevalence. Anemia was slightly higher among those with a history of blood transfusion (65.98%) compared to those without (60.55%), though not significant (*p* = 0.3). Similarly, no significant associations were found for sickle cell disease/trait, thalassemia (*p* = 0.1), or worm infestation (*p* = 0.7). Although 68.85% of participants were enrolled in government nutritional supplementation schemes, this did not significantly influence anemia prevalence (*p* = 0.5). However, Iron and Folic Acid (IFA) supplementation showed a significant relationship: girls who did not consume IFA tablets had a higher prevalence of anemia (*p* = 0.02). Moreover, lack of knowledge about IFA was significantly associated with anemia (*p* = 0.004). Frequency or timing of IFA intake did not demonstrate significant associations (Supplementary Tables S1, S2).

### 3.4 Anemia knowledge and awareness

A strong inverse relationship was observed between knowledge score and anemia prevalence (*p* < 0.001). Girls with low knowledge scores (0–3) had the highest prevalence (66.3%), whereas those with moderate scores (4–6) had 60.8%, and those with high knowledge scores (7–9) showed a markedly lower prevalence (18.5%). These findings emphasize the role of awareness and health education in mitigating anemia burden among adolescents (Table 3).

**Table 3:** Association of overall knowledge score regarding anemia with anemia prevalence in adolescent girls .

Overall knowledge (education score)^a^ regarding anemia	Anemia (*n*, row %, Col %)	Not anemia (*n*, row %, Col %)	Raw total (*n*, %)	*P* value
Low (0–3)	1,101 (66.3%, 73.7%)	559 (33.7%, 44.8%)	1,660 (59.5%)	<0.001
Moderate (4–6)	394 (60.8%, 26.4%)	254 (39.2%, 20.4%)	648 (23.2%)	
High (7–9)	92 (18.5%, 6.2%)	405 (81.5%, 32.5%)	497 (17.8%)	
Column Total	1,497 (53.7%)	1,248 (46.3%)	2,805 (100%)	

a The overall knowledge score was calculated based on nine binary (Yes/No) questions assessing awareness and understanding of anemia: (1) “Have you heard of anemia?” (2) “Do you know what causes anemia?” (3) “Do you know common symptoms?” (4) “Do you know prevention/treatment methods?” (5) “Are you aware of iron's dietary importance?” (6) “Can you list iron's roles in the body?” (7) “Do you know iron-rich foods?” (8) “Are you aware of IFA tablets?” and (9) “What is IFA's importance?” Each “Yes"/correct answer received 1 point (max score = 9), categorized as Low (0–3), Moderate (4–6), or High (7–9) knowledge. The score demonstrated good internal consistency (Cronbach's α = 0.78) and was adjusted for education/socioeconomic status in analyses.

### 3.5 Comparison of hematological and biochemical parameters

Hematological indices demonstrated marked differences: hematocrit, MCV, MCH, and MCHC were all significantly lower among anemic participants (*p* < 0.001), while RDW-CV and reticulocyte counts were significantly elevated (*p* < 0.001). Biochemical parameters also showed consistent trends: anemic girls had significantly lower serum iron (64.0 µg/dL vs. 86.0 µg/dL), ferritin (11.1 ng/mL vs. 17.5 ng/mL), and transferrin saturation (14.73% vs. 20.76%), with higher TIBC (445.85 µg/dL vs. 425.24 µg/dL) and transferrin (343.08 mg/dL vs. 327.09 mg/dL) levels (*p* < 0.001), indicating iron deficiency anemia. Serum albumin and prealbumin were also lower among anemic girls, whereas CRP levels were significantly elevated (*p* < 0.001), suggesting an inflammatory component. No significant differences were observed in Vitamin B12, folate, or Vitamin D levels between groups (Supplementary Table S3).

### 3.6 Hemoglobin variant analysis and iron deficiency status

**Figure 2:**
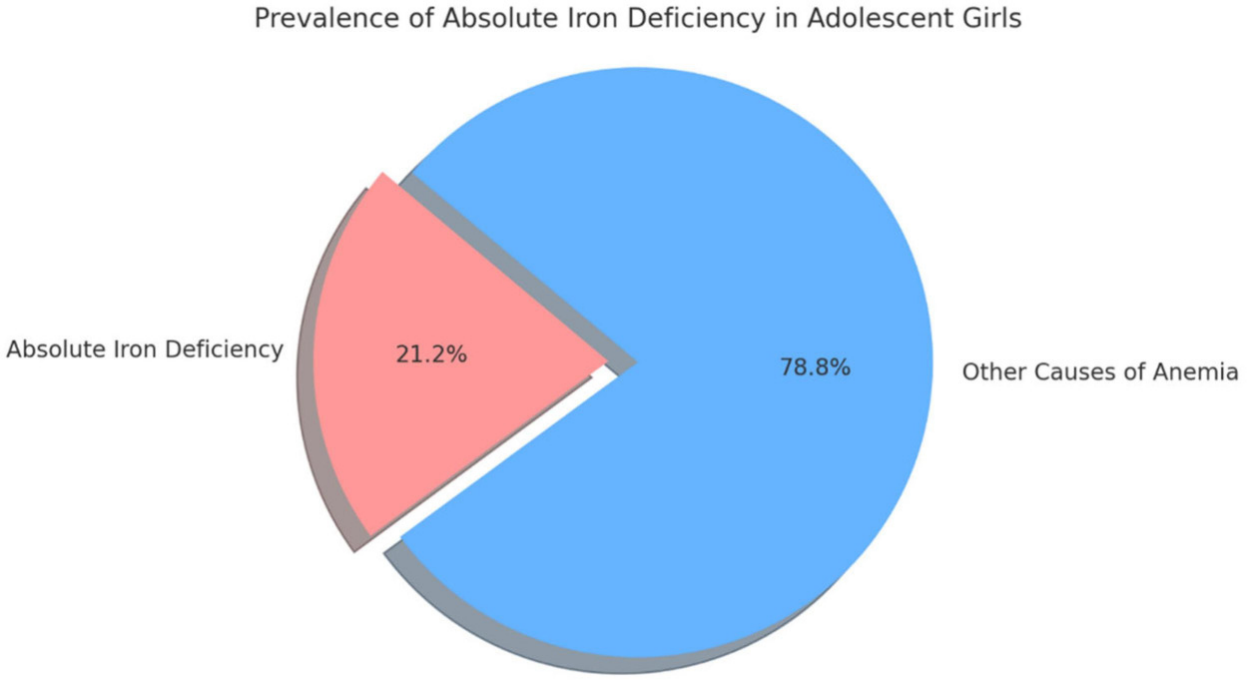
Prevalence of absolute iron deficiency in adolescent girls.

Based on iron profile assessment, absolute iron deficiency was detected in 21.19% (*n* = 363) of anemic girls, whereas 78.81% (*n* = 1350) had anemia due to other etiologies such as chronic disease or hemoglobinopathies (Figure 2). Among 791 anemic girls who underwent High-Performance Liquid Chromatography (HPLC) testing—performed selectively based on abnormal peripheral smear findings—86.8% showed a normal HPLC pattern. Among abnormal findings, Beta Thalassemia Trait (5.4%) was most common, followed by Sickle Cell Trait (4.8%), Codominant (Sickle + Beta Thalassemia) (2.4%), and HbD Disease/Trait (0.5%), reflecting the regional hemoglobinopathy spectrum (Table 4).

**Table 4:** Prevalence of haemoglobin variants in adolescent anemic girls.

Hb Variant	Number of cases (*n*)	Prevalence (%)
Normal HPLC	687	86.9%
Beta Thalassemia Trait	43	5.4%
Sickle Cell Trait	38	4.8%
Codominant (Sickle + Beta Thalassemia)	19	2.4%
HbD Disease/Trait	4	0.5%
Total anemia cases underwent HPLC analysis	791^a^	100.0%

a HPLC testing (*n* = 791) was selectively performed on anemic adolescent girls with abnormal peripheral smear findings to prioritize hemoglobinopathy detection.

Multivariate logistic regression was done to identify the independent predictors of anemia among adolescent girls with statistically significant variables (*p* < 0.05) in bivariate analyses. On multivariate logistic regression, the status of family health coverage (OR: 1.151, 95% CI: 1.046–1.267, *p* = 0.004) was significantly associated with anemia among adolescent girls. This indicates that study participants with health insurance had 15.1% higher odds of being anemic compared to those without health insurance as those with health insurance has more odds of seeking healthcare and thus higher odds of anemia detection compared to their counterpart. Though availability of electronic devices has an odds ratio of 1.073, it is not statistically significant. Family belonging to BPL status wasn't significantly associated with anemia (OR: 0.995, 95% CI: 0.898–1.102, *p* = 0.923). The overall knowledge/education score regarding anemia among the study participants has an marginal significant with OR: 0.892, 95% CI: 0.790–1.007, *p* = 0.062 (Table 5).

**Table 5:** Multivariate logistic regression between anemia status and different variables.

Variable	aOR	SE	95% CI	*p* value
Family Below Poverty Line	0.995	0.052	0.898–1.102	0.923
Health insurance coverage	1.151	0.049	1.046–1.267	0.004^*^
Using mobile/computer/laptop/tablet	1.073	0.046	0.981–1.173	0.124
Overall knowledge regarding anemia	0.892	0.062	0.790–1.007	0.064

aOR, adjusted odds ratio; SE, standard error; CI, confidence interval. Reference categories: Above Poverty Line (for family income status); No health insurance coverage; Not using mobile/computer/laptop/tablet; Poor knowledge regarding anemia.

*statistically significant *p*-value.

## 4 Discussion

This study shows a detailed analysis of prevalence of anemia among adolescent girls residing in the state of Gujarat. 2815 adolescent girls from 10 selected districts of the state were included in the study and a detailed study on prevalence of anemia among the study population and various sociodemographic and nutritional factors, dietary and hygiene habits, biochemical and hematological parameters were studied. In addition, knowledge on anemia among adolescent girls with anemia was also studied. Higher anemia prevalence despite various governmental strategies including the Anemia Mukt Bharat is itself a testimony that our interventions still need refinement. In this context, this study helps in identifying various factors associated with anemia prevalence among the adolescent girls.

The overall prevalence of anemia among the study population was 60.85%, which is more than half of the global burden as estimated by WHO (29.9%) (16) and is almost similar to that reported in NFHS—5 survey (59%) (4). This suggests that the anemia burden persist in spite of various actions implemented by the government. Moreover, a nationwide systematic review and meta-analysis of 35 community-based studies comprising 152,640 participants reported a pooled prevalence of 65.7% among adolescent girls (17). Comparative studies done around the globe in Sub-Saharan Africa, Nepal and Indonesia shows the prevalence of anemia among adolescent girls ranging from 14% to 68% with lowest being reported in Indonesia and highest in Nepal (18–20).

Among the 10 districts included in the study, we noticed a wide range of inter-district variation ranging from 42.37% in Rajkot Municipal Corporation to 75.00% in Sabarkantha district. Highest prevalence of anemia was reported among the districts of Dahod, Tapi and Navsari which are predominantly comprising tribal population and thus raising a concern on the vulnerability of tribal population to anemia and other nutritional services. This finding aligns with the prevalence of anemia among tribal adolescent girls which is 87.5% as stated by the nationwide comparative studies (17).

The study reports 33.4% and 25% of the participants have mild and moderate anemia which is similar to that found in the study done in the western Maharashtra (21). The NFHS—5 reports also substantiate this finding as it reports the prevalence of mild and moderate anemia among adolescent girls as 26% and 30% respectively (4).

We found that anemia was more prevalent among tribal population (73.57%) followed by rural (56.39%) and urban population (48.19%) has the least among the subgroups. This finding is well supported by intra-state regional variation of anemia prevalence documented in the Comprehensive Nutrition Survey in Gujarat (CNSG), 2019 (7).

The socioeconomic status of the family influences the anemia prevalence to an extent as evident from the study finding which reports that the prevalence of anemia among adolescent girls belonging Below Poverty Line (BPL) families was 61.68% compared to 56.57% in those belonging to non-BPL families. The study conducted using the DLHS—4 data also documented similar observation of lower the socioeconomic status, higher is the prevalence of anemia (22). This difference is majorly attributed to the dietary habits, healthcare accessibility and awareness about anemia and its prevention.

This study finds that anemia prevalence has an association with accessibility to digital connectivity like mobile phones, laptops, computer or tablet. It is evident from the finding that the prevalence of anemia among those who lack digital connectivity which is 65.41% drops to 58.66% among those who have access. This is a significant finding as digital accessibility can by-pass the socioeconomic status and awareness levels and can act as key tool for implementing mobile health interventions in underserved populations like tribal populations. Study conducted with NFHS—5 data also suggests that media exposure is one of the significant factors associated with anemia prevalence (23).

Other sociodemographic features studied includes Age, education level, type of houses, type of family, religion and caste. The association of these factors with anemia prevalence is not statistically significant, but more prevalence is seen with those in the age group of 15–19 years, having studied up to secondary education, those living in semi-pucca houses and in a joint family and belonging to Hindu religion.

The study documents significant association between IFA supplementation and knowledge about IFA supplementation and anemia prevalence among adolescent girls. This is well supported by a similar study done among the school going children in Karnataka which documented low prevalence (51%) among children on WIFS compared to those who are not provided with WIFS (64.4%) (24). Similarly, in a study done in Nagpur reported no significant difference in improvement of haemoglobin post daily and weekly IFA supplementation (1.04 ± 0.7 g/dL in the daily group vs. 0.95 ± 0.8 g/dL in the weekly group) but the researchers have identified better compliance with drug intake when it is provided weekly (25).

Though we have developed delivery systems for WIFS, IFA supplementation alone will not bring about the change we aspire. It is the behavioural change along with IFA supplementation is the one needed to address the anemia burden. This is significant from our study which finds that there is an inverse relationship between knowledge score and anemia prevalence. We calculated the knowledge score with a questionnaire and all the participants were tested with it. Girls with lowest scores (0–3) has the higher anemia prevalence (66.3%) compared to those with high scores (7–9) in whom the prevalence is 18.5%.

This is well supported by the results of a qualitative study on anemia prevalence among adolescents and their perception about anemia in Varanasi which shows the study population demonstrated poor knowledge, inadequate awareness and effects of the disease (26). Similar findings are also noted in a study in Vietnam where reduced anemia rate was seen among those who have knowledge about iron-rich foods (AOR = 0.60, 95% CI: 0.44–0.83) (27). These findings implies the critical role of Weekly Iron and Folic acid Supplementation programme (WIFS) and related health education and awareness activities to reduce the anemia burden in the community. Thus, integrating health education into school curriculum needs more attention to promote awareness thereby leading to more positive attitude towards the disease prevention and thus achieving the ultimate goal of disease burden reduction.

There was also a significant inverse association between beverages consumption like tea or coffee or both and anemia prevalence among the study population. Higher prevalence was reported among those who consume both tea and coffee (69.1%) followed by those who consume tea (61.0%) compared to the non-consumers (60.3%). This can be due to the fact that tea and coffee contain tannins and polyphenols respectively which chelate with the non-heme iron and thus reducing the absorption of iron in the dietary intake by up to 60% (28, 29). Study conducted in Delhi documented that >97% of the adolescents participated in the study drink tea or coffee around meal (30). Dietary habits and its diversity across the region especially inadequate intake of iron-rich foods and Vitamin C along with poor sanitation and hygiene practices is one of the critical factors associated with iron deficiency among adolescent girls as stated by the CNNS 2016–18 (7). Similarly, a study on Ethiopian adolescent girls to find prevalence of anemia and its associated factors had reported that consumption of tea/coffee within 30 min after meals as one of the independent predictors of anemia prevalence (31).

We have found that there is no significant association between BMI and anemia prevalence but height and weight of the adolescent girls have a significant association individually. Lower body weight was observed among adolescent girls with anemia (40.84 ± 9.29) when compared with non-anemic adolescents (41.57 ± 9.78). This similar finding was reported in a study done among school going adolescent girls in rural areas of selected districts of Maharashtra reported 74.2% of underweight girls had anemia followed by normal weight, overweight and obese girls (32). Another study done in Maharashtra among adolescent girls in rural areas reported increase in haemoglobin levels with increase in each of height, weight and Mid Upper Arm Circumference (MUAC) of an individual (33). Similarly, in Nepal it has been reported that anemia among adolescent school going girls was more prevalent among underweight girls (20.7%), followed by normal weight (16.6%) and overweight (15.1%) (19). and study done among sub-Saharan Africa population also reported significant association between underweight status and anemia prevalence (18).

Our study reported the classical laboratory findings in the case of iron deficiency anemia which shows reduced serum iron, ferritin, and transferrin saturation and elevated Total Iron Binding Capacity in anaemic individuals compared to that in non-anaemic. This classic laboratory findings aligns with the CNNS 2016–18 report which states that reduced serum ferritin (<15 µg/L) is a sign of iron deficiency and thus is a strong predictor of anemia among Indian adolescents (7).

Inflammation increases hepcidin production, inhibits ferroportin, reduces iron absorption and induce oxidative stress and thereby interfering iron absorption (34). Studies have reported that inflammation alone or in combination with iron deficiency accounts for anemia prevalence worldwide up to 40% (35, 36). The study reports significantly elevated C-Reactive Protein (CRP) among anemic individuals suggesting role of inflammation in anemia etiology. Several studies reported that there is a significant inverse association between CRP and Hemoglobin level that is higher the CRP levels, lower is the hemoglobin values. The study done in Indonesia among adolescent girls showed higher CRP values among anemic adolescents (37). Similarly, a study on determinants of anemia among young children in rural areas of Karnataka also showed raised CRP levels had an association with lower hemoglobin levels (38). Thus, our finding of significant inverse association between CRP and hemoglobin levels can overcome the diagnostic difficulties in interpretation of serum ferritin levels in an inflammatory state. In addition to CRP levels, estimation of serum Hepcidin level will also be helpful in diagnosis of mild anemic cases as studies found significant occurrence of elevated serum hepcidin and CRP levels in mild anemic cases (39, 40).

Haemoglobin variant analysis done among anaemic girls in our study reveals Beta Thalassemia Trait (5.44%), Sickle Cell Trait (4.80%), and Codominant Sickle-Beta Thalassemia (2.40%). This finding shows that the state of Gujarat is within the Thalassemia belt. In India, prevalence of Beta-thalassemia trait is almost 3%–4%, translating to about 42 million carriers (41) and our findings aligns with this report. In a systematic review and meta-analysis of 87 studies studying on the prevalence of Sickle cell disease, Sickle cell trait and HBS-beta-thalassemia in India, it has been found that the sickle cell and thalassemia traits have been most prevalent among the tribal population with frequency ranging from 1% to 40% depending on the various subgroups (42). This finding was marginally substantiated by a regional study done in the state of Andhra Pradesh, which reported Sickle cell trait and beta-thalassemia trait were prevalent among 65.5% and 1.51% respectively (43). The identification of hemoglobinopathies is as important as identifying iron deficiency, since hemoglobinopathies are the second most common predictor of anemia with OR: 2.81 (95% CI: 1.66–4.74) with iron deficiency being the most common and leading predictor as reported by the Comprehensive National Nutrition Survey (CNNS) 2016–2018 (7). All these findings suggests integration of hemoglobinopathy screening with anemia screening especially in a hemoglobinopathy prevalent regions like Gujarat.

Lower socio-economic status is known to be associated with prevalence of nutritional anemia (4) but in the logistic regression, it is not significant due to the confounding by factors like health insurance coverage and knowledge score. Health insurance coverage is significantly associated with anemia prevalence with an OR 1.151 (95% CI: 1.046–1.267, *p* = 0.004) which proves that health insurance coverage does serve as a proxy for healthcare access as insured girls have higher chance for access to routine screening thus increasing the odds of diagnosis of anemia, thereby increasing its prevalence. This finding was well supported by a systematic review on the impact of public health insurance schemes on healthcare utilisation in India (44). Though access to electronic devices is not significantly associated with anemia prevalence in our study, use of digital interventions in improving health awareness and service delivery has been strongly recommended by the WHO (45). Knowledge about anemia showed marginal significance with anemia prevalence in our study. Various studies suggest low knowledge levels were significantly associated with increased anemia prevalence (46) and also knowledge level alone can act as independent predictor of anemia prevalence (47). But knowledge level and anemia prevalence is not always intuitive. A study done in Indonesia demonstrated that good knowledge was observed among anaemic adolescent girls which can be explained by the fact that those diagnosed might read more about the disease (48) whereas another study showed parental support was an important predictor than knowledge levels in predicting adolescent anemia (49).

These findings should be interpreted with caution in light of certain limitations. First, the cross-sectional study design limits the ability to establish causal relationships between the associated factors and anemia outcomes. Additionally, the use of self-reported information for dietary intake, iron–folic acid supplementation, and hygiene practices may have introduced recall or social desirability bias. In addition, the present analysis was primarily descriptive and based on bivariate comparisons; multivariable regression modelling to estimate adjusted effect sizes and account for potential confounders or clustering effects was not performed. Future analyses using this dataset are planned to explore independent predictors of anemia through multivariable and hierarchical modelling approaches. Although several key biochemical markers were assessed, the inclusion of additional indicators such as hepcidin or interleukins could have provided deeper mechanistic insights. Finally, the lack of longitudinal follow-up restricts our understanding of how anemia evolves over time and the potential impact of ongoing interventions.

## 5 Conclusion

This multicentric study reveals a high burden of anemia among adolescent girls, affecting nearly two-thirds of participants, with clear disparities in tribal and rural populations reflecting underlying social inequities. The findings indicate that anemia is not solely driven by iron deficiency, emphasizing the need to move beyond universal supplementation toward a stratified approach that includes screening, diagnostic evaluation, and management of non–iron deficiency causes such as inflammation and hemoglobinopathies. The strong association between higher knowledge and lower anemia prevalence highlights the importance of adolescent-focused health education and improved IFA adherence. Policy efforts should prioritize high-burden regions, strengthen adolescent health services, integrate hemoglobinopathy screening in endemic areas, and address broader determinants such as health literacy and access to resources. The limited impact of current supplementation programs calls for better monitoring and program redesign. Overall, reducing adolescent anemia requires a multisectoral, equity-focused strategy that combines nutrition, education, early diagnosis, and strengthened health systems.

## Data Availability

The raw data supporting the conclusions of this article will be made available by the authors, without undue reservation.
