# Supplementary material for: Factors associated with anemia among adolescent girls in Western India: insights from a multi-centric cross-sectional study
Source: Front Glob Womens Health. 2026 May 18;7:1793809. doi: 10.3389/fgwh.2026.1793809 (PMC13222952; doi:10.3389/fgwh.2026.1793809)
Supplement: Supplementary file 1 [file Table1.docx]

**Supplementary Table 1. Association of Medical/Nutritional intervention and IFA Supplementation Factors with prevalence of anemia among adolescents**

| Variables | **Anemia status among Adolescent girls** | | | P value |
| --- | --- | --- | --- | --- |
|  | **Anemia**  (n, Row %, Col %) | **Not Anemia**  (n, Row %, Col %) | Raw Total  (n, %) |  |
| **Blood transfusion any time in life** | | | | |
| No response | 28(68.29%, 1.63%) | 13(31.71%, 1.18%) | 41 (1.46 %) | 0.3 |
| No | 1621(60.55%, 94.63%) | 1056(39.45%, 95.83%) | 2677 (95.10%) |  |
| Yes | 64(65.98%, 3.74%) | 33(34.02%, 2.99%) | 97 (3.45%) |  |
| Column Total (n, %) | 1713(60.85%) | 1102(39.15%) | 2815 (100%) |  |
| **History of Worm infestations** | | | | |
| Don’t know | 160 (63.24%, 9.34%) | 93 (36.76%, 8.44%) | 253 (8.99%) | 0.7 |
| No | 1475 (60.57%,86.11%) | 960 (39.43%, 87.11%) | 2435 (86.50%) |  |
| Yes | 78 (61.42%, 4.55%) | 49 (38.58%, 4.45%) | 127 (4.51%) |  |
| Column Total (n, %) | 1713 (60.85%) | 1102 (39.15%) | 2815 (100%) |  |
| **Habit of consuming unusual items (non-food items)** | | | | |
| No | 1619 (60.98%, 94.51%) | 1036 (38.02%,94.01%) | 2655 (94.32%) | 0.5 |
| Yes | 94 (58.75%, 5.49%) | 66 (41.25%, 5.99%) | 160 (5.68%) |  |
| Column Total (n, %) | 1713 (60.85%) | 1102 (39.15%) | 2815 (100%) |  |
| **Avail nutritional scheme** | | | | |
| Don’t know | 5 (71.43%, 0.29%) | 2 (28.57%, 0.18%) | 7 (0.25%) | 0.5 |
| No | 517 (59.43%, 30.18%) | 353 (40.57%, 32.03%) | 870 (30.91%) |  |
| Yes | 1191(61.46% 69.53%) | 747 (38.54%, 67.79%) | 1938 (68.85%) |  |
| Column Total (n, %) | 1713 (60.855) | 1102 (39.15%) | 2815 (100%) |  |

**Supplementary Table 2. Association between IFA supplementation and anemia prevalence in adolescent girls**

| Variables | **Anemia status among Adolescent girls** | | |  |
| --- | --- | --- | --- | --- |
|  | **Anemia**  (n, Row %, Col %) | **Not Anemia**  (n, Row %, Col %) | Raw Total  (n, %) | P value |
| **Advised to take IFA tablets** | | | | |
| No | 32 (46.38%) | 37 (53.62%) | 69 (29.61%) | 0.447 |
| Yes | 85 (51.83%) | 79 (48.17%) | 164 (70.39%) |  |
| Column Total (n, %) | 117 (50.21%) | 116 (49.79%) | 233 (100%)* |  |
| **In the last month, how many times did you consume Iron & Folic acid tablet?** | | | | |
| did not consume | 134 (56.78%, 9.94%) | 102(43.22%, 2.07%) | 236 (10.76%) | 0.47 |
| occasionally | 74 (59.68%,5.49%) | 50 (40.32%,5.92%) | 124 (5.65%) |  |
| 3 days every week | 191 (63.67%,14.17%) | 109(36.33%,12.90%) | 300 (13.68%) |  |
| 4 or more days every week | 69 (58.97%5.12%) | 48 (41.03%,5.92%) | 117 (5.34%) |  |
| once every week | 880 (62.15%,65.28%) | 536(37.85%%,63.43%) | 1416 (64.57%) |  |
| Column Total (n, %) | 1348(61.47%) | 845(38.53%) | 2193 (100%)* |  |
| **When & with what do you consume Iron & Folic acid?** | | | | |
| After/with meal with lemon juice | 29 (50.88%, 2.39%) | 28 (49.12%, 3.77%) | 57 (2.91%) | 0.07° |
| After/with meal with milk/tea/ coffee | 5 (38.46%, 0.41%) | 8 (61.54%, 1.08%) | 13 (0.66%) |  |
| After/with meal with water | 987 (61.92%, 81.30%) | 607 (38.08%, 81.70%) | 1594 (81.45%) |  |
| Before meal with lemon juice | 2 (50.00%, 0.16%) | 2 (50.00%, 0.27%) | 4 (0.20%) |  |
| Before meal with milk/tea/coffee | 7 (87.50%, 0.58%) | 1 (12.50%, 0.13%) | 8 (0.41%) |  |
| Before meal with water | 184 (65.03%, 15.16%) | 97 (34.52%, 13.06%) | 281 (14. 36%) |  |
| Column Total (n, %) | 1214 (62.03%) | 743 (37.97%%) | 1957(100.00%)* |  |
| **Are you facing any side effects for consuming IFA tablets?** | | | | |
| No | 1094(62.09%,90.12%) | 668(37.91%,89.91%) | 1762 (90.04%) | 0.8 |
| Yes | 120 (61.54%,9.88%) | 75 (38.46%,10.09%) | 195(9.96%) |  |
| Column Total (n, %) | 1214(62.03%) | 743(37.97%) | 1957 (100%)* |  |

°Fischer Exact Test

*Missing values in certain variables (e.g., some participants did not respond or selected "don’t know").

*Filtered analysis applied to specific questions where valid data was available only for a subset of the total population.

**Supplementary table 3.** **Comparison of demographics, anthropometrics, clinical, hematological, and biochemical parameters between anemic and non-anemic adolescent girls**

| **Parameter** | **Anemia**  **Mean ± SD or**  **Median (IQR)***  **[n= 1713]** | **Non-Anemia**  **Mean ± SD or**  **Median (IQR)***  **[n=1102]** | **P value** |
| --- | --- | --- | --- |
| **Age (Years)** | 15.13 ± 2.12 | 15.17 ± 2.18 | 0.58 |
| **Height (m)** | 1.49 ± 0.08 | 1.50± 0.08 | 0.03 |
| **Weight (kg)** | 40.84 ± 9.29 | 41.57 ± 9.78 | 0.04 |
| **BMI (kg/m²)** | 18.22±3.99 | 18.35±4.05 | 0.40 |
| **SBP (mmHg)** | 109.42±12.13 | 109.42±12.04 | 0.99 |
| **DBP (mmHg)** | 70.03±0.03 | 69.78±9.07 | 0.47 |
| **WBC (x10³/µL)** | 7.6 (2.49) * | 7.74 (2.46) | 0.06 |
| **Neutrophils (%)** | 53.97±13.90 | 52.86±15.48 | 0.05 |
| **Lymphocytes (%)** | 34.9 (12.1) * | 35.9 (12.2) * | **< 0.001^** |
| **Monocytes (%)** | 7 (2.6)* | 6.9 (2.6)* | 0.007 |
| **Eosinophils (%)** | 3.77±4.36 | 3.39±3.2 | 0.01 |
| **Basophils (%)** | 0.4 (0.3)* | 0.4 (0.3)* | <**0.001^** |
| **RBC (millions/µL)** | 4.71±0.63 | 4.73±0.59 | 0.37 |
| **HCT (%)** | 36.29±3.71 | 41.00±3.12 | **< 0.001** |
| **MCV (fL)** | 77.97 ± 10.59 | 87.10 ± 8.11 | **<0.001** |
| **MCH (pg)** | 23.20±4.63 | 27.17±3.48 | **< 0.001** |
| **MCHC (g/dL)** | 29.53±2.16 | 31.12±1.80 | **< 0.001** |
| **RDW-CV (%)** | 15.5 (2.9)* | 13.7 (1.5)* | **<0.001^** |
| **Platelets (x10³/µL)** | 350.03±101.02 | 337.39±85.79 | **< 0.001** |
| **Reticulocytes (%)** | 0.8 (0.2)* | 0.6 (0.2)* | **<0.001^** |
| **HbA2 (%)** | 2.7 (0.6)* | 3 (0.5)* | 0.21 |
| **HbF (%) {n=269}** | 0.2 (0.3)* | 0.2 (0.18) * | 0.29 |
| **Iron (µg/dL)** | 64.0 (53.0)* | 86.0 (44.0)* | **<0.001^** |
| **Magnesium (mg/dL)** | 1.91±0.13 | 1.92±0.13 | 0.42 |
| **CRP (mg/L)** | 4.0 (10.5)* | 4.0 (2.9)* | **<0.001^** |
| **Albumin (g/dL)** | 4.55±0.31 | 4.63±0.32 | **< 0.001** |
| **Ferritin (ng/mL)** | 11.1 (13.36)* | 17.5 (15.7)* | **<0.001^** |
| **Vitamin B12 (pg/mL)** | 200.00 (92.00)* | 200.55 (88.00)* | 0.42 |
| **Vitamin D (ng/mL)** | 22.3 (10.8)* | 21.7 (10.1)* | 0.66 |
| **Transferrin (mg/dL)** | 343.08 ±55.18 | 327.09±46.95 | **< 0.001** |
| **Transferrin Saturation (%)** | 14.73 (13.82) * | 20.76 (11.68)* | **< 0.001^** |
| **TIBC (µg/dL)** | 445.85 ± 62.41 | 425.24 ± 51.55 | **< 0.001** |
| **Folate (ng/mL)** | 4.18 (2.62)* | 4.14 (3.16)* | 0.95 |
| **Prealbumin (mg/dL)** | 19.28±3.59 | 20.08±3.82 | **< 0.001** |

P-value calculated using independent t-test and ^^^Mann-Whitney U test
